# Racial differences in skeletal fragility but not osteoarthritis among women and men with cerebral palsy^☆^

**DOI:** 10.1016/j.bonr.2019.100219

**Published:** 2019-07-30

**Authors:** Daniel G. Whitney

**Affiliations:** Department of Physical Medicine and Rehabilitation, University of Michigan, 325 E. Eisenhower, Ann Arbor, MI 48108, United States of America; Institute for Healthcare Policy and Innovation, University of Michigan, 2800 Plymouth Rd., Ann Arbor, MI 48109, United States of America

**Keywords:** CP, cerebral palsy, Cerebral palsy, Osteoporosis, Fracture, Osteoarthritis, Clinical epidemiology

## Abstract

**Background:**

Adults with cerebral palsy (CP) have increased risk for skeletal fragility and osteoarthritis. However, racial differences in these outcomes have not been examined. Such knowledge could improve patient-specific clinical care for the prevention and management of these conditions. The purpose of this study was to determine if there were racial differences in the prevalence of osteoporosis, all-cause fracture, and osteoarthritis among young and middle-aged adults with CP.

**Methods:**

Data from 2016 were extracted from a random 20% sample of the Medicare fee-for-service database. International Classification of Diseases, Tenth Revision, Clinical Modification codes were used to identify 18–64 year olds with CP, as well as osteoporosis, all-cause fracture, osteoarthritis, and neurodevelopmental and noncommunicable disease comorbidities.

**Results:**

Of the 16,488 adults with CP, 13,334 were White, 2477 were Black, and 677 were Hispanic. The age-standardized prevalence of osteoporosis (women: 12.9%, 9.0%, 8.3%, respectively; men: 9.2%, 4.8%, 7.9%, respectively) and fracture (women: 7.4%, 4.2%, 9.9%; men: 6.0%, 2.3%, 6.0%) was lower for Black adults with CP compared to White adults with CP (all *p* < 0.005). No racial differences were observed for age-standardized prevalence of osteoarthritis (women: 13.6%, 14.4%, 9.6%; men: 10.7%, 10.4%, 12.7%). The racial differences between Black and White adults with CP remained even after adjusting for age, U.S. region, neurodevelopmental comorbidities, and several noncommunicable diseases for osteoporosis (women: OR = 0.66, 99.5% CI = 0.48–0.91; men: OR = 0.51, 99.5% CI = 0.35–0.75) and fracture (women: OR = 0.57, 99.5% CI = 0.37–0.89; men: OR = 0.39, 99.5% CI = 0.23–0.68).

**Conclusions:**

Study findings suggest racial differences in skeletal fragility among young and middle-aged adults with CP, with White women and men with CP having greater risk compared to Black women and men with CP. This study found no evidence of racial differences in the prevalence of osteoarthritis.

## Highlights

• Racial differences for skeletal fragility and osteoarthritis among adults with cerebral palsy (CP)have not been investigated
• Prevalence of osteoporosis and fracture, but not osteoarthritis, was higher for White vs. Black women and men with CP
• No racial differences were observed for Hispanic vs. White or Black women and men with CP
• Racial differences in skeletal fragility were present even after adjusting for potential confounding factors

## 1 Introduction

Cerebral palsy (CP) is the most common physical disability of childhood and affects about 3.1 per 1000 children in the U.S. (Christensen et al., 2014). The etiology of CP and the resulting clinical phenotype varies, encompassing genetic predisposition, brain lesions, and maternal/environmental exposures leading to damage or malformation of the developing brain (Villamor et al., 2017). While the health, function, and medical profiles are complex and heterogeneous, what links all individuals with CP is some degree of motor impairment, risk for restriction in activities of daily living, and low societal integration. Collectively, these factors increase susceptibility for poor growth and development, especially of the musculoskeletal system.

Children with CP have low levels of physical activity (Whitney et al., 2017; Johnson et al., 2009) and an underdeveloped musculoskeletal system (Whitney et al., 2017; Johnson et al., 2009; Modlesky et al., 2015) compared to typically developing children, regardless of the severity of CP. The result is a skeletally fragile phenotype in the trabecular (Modlesky et al., 2015) and cortical (Whitney et al., 2017; Modlesky et al., 2009) bone compartments with overall bone strength deficits ranging from 34% (milder forms of CP) (Whitney et al., 2017) to 71% (moderate-to-severe forms of CP) (Modlesky et al., 2009) compared to age, sex, and race matched typically developing children. Further, children with CP have elevated musculoskeletal (Whitney et al., 2017), body (Whitney et al., 2019a), and abdominal (Whitney et al., 2018a) fat, which may further impede musculoskeletal development independent of mechanical loading (Whitney et al., 2018b), leading to exacerbation of skeletal fragility and excess joint stress as children with CP age into and throughout their adult years. Indeed, in the first stage of adulthood (18–30 years), individuals with CP are 7 times more likely to have a musculoskeletal morbidity, including osteoporosis and osteoarthritis, compared to individuals without CP after controlling for potential confounding factors (Whitney et al., 2018c). Further, the prevalence of osteoporosis and osteoarthritis becomes more pronounced throughout the lifespan for individuals with CP, with the prevalence of osteoporosis being >2.5 times higher among >50 year olds with CP (Whitney et al., 2018d) compared to the general population > 50 years old (Wright et al., 2014). All-cause fracture is also >2 times higher among young and middle-aged adults with CP compared to young and middle-aged adults without CP, with the increased fracture risk present even after accounting for chronic diseases and osteoporosis (Whitney et al., 2019b).

Osteoporosis, fracture, and osteoarthritis are high-burden conditions and negatively impact health (Cristancho et al., 2016; Veronese et al., 2017; Misra et al., 2015), survival (Neuman et al., 2014; Brauer et al., 2009; Kumar et al., 2018; Rapp et al., 2010; Uriz-Otano et al., 2016), and quality of life (Salkeld et al., 2000; Harvey-Kelly et al., 2014; Murphy et al., 2016; Xie et al., 2016), and account for a substantial economic burden (Xie et al., 2016; Burge et al., 2007). When considering the recent literature regarding the magnitude of the problem of these conditions for adults with CP (Whitney et al., 2018c; Whitney et al., 2018d; Whitney et al., 2019b; O'Connell et al., 2019), recommendations for earlier screening and preventive strategies have been made (Whitney et al., 2019b). However, epidemiologic studies are lacking for determining if racial differences exist for skeletal fragility (i.e., osteoporosis and fracture) and osteoarthritis among adults with CP, despite the well-documented literature on racial differences within the general population for these conditions. Evidence suggests racial differences in the prevalence of and risk factors for CP (e.g., birth weight) (Wu et al., 2011). Racial differences in developing CP and the resulting health complications experienced throughout the lifespan may stem from behavioral factors (e.g., pre- and post-natal care), institutional factors (e.g., healthcare disparities), socioeconomic factors (e.g., neighborhood poverty) (Messer et al., 2008), psychosocial stressors (Dailey, 2009), genetics (Menon et al., 2006), and epigenetics (Crowgey et al., 2018; Mohandas et al., 2018), which are all associated with the pathophysiology of skeletal fragility and osteoarthritis. Taken together, individuals with CP may have a unique interplay between genetic, environmental, and behavioral constructs that lead to early development of skeletal fragility and osteoarthritis, which may be further influenced by race. However, little research attention has been given to investigating racial differences in health outcomes for this underserved adult population. By knowing racial differences in skeletal fragility and osteoarthritis, clinicians treating adults with CP may be better equipped to provide clinical care by adopting earlier screening, treatment, and preventive strategies unique to their patient to optimize skeletal and joint health throughout the lifespan. Therefore, the primary objective of this study was to determine if there were racial differences among young and middle-aged White, Black, and Hispanic individuals with CP for the prevalence of osteoporosis, all-cause fracture, and osteoarthritis.

## 2 Methods

### 2.1 Data source

Data were extracted from a random 20% sample of the Medicare fee-for-service database from the Centers for Medicare and Medicaid Services (CMS). Claims data from the Medicare Provider Analysis and Review, Outpatient, and Carrier files (Parts A and B), which are primarily used for reimbursement purposes, were leveraged from the year 2016. Pharmacy claims were not available for this study. Eligibility for Medicare enrollment in 2016 includes all individuals 65 years of age and older and individuals with disabilities at any age and of any income status. Since the data are de-identified, the local Institutional Review Board approved this study as non-regulated.

### 2.2 Sample selection

International Classification of Diseases, Tenth Revision, Clinical Modification (ICD-10) codes were used to identify all medical conditions. ICD-10 codes are entered into the billing system by health service providers. Information regarding how diagnoses were made (e.g., DXA) or by whom (e.g., primary care provider) are not available. Individuals that were covered by Health Maintenance Organization plans were excluded because of incomplete claims, thus biasing estimates. Inclusion criteria for this study were: at least one claim in any position for any CP diagnosis (G80 family codes; spastic hemiplegia, spastic diplegia, spastic quadriplegia, athetoid, ataxic, and other/unspecified CP); between 18 and 64 years of age; 12 months of continuous enrollment in a health plan in 2016; at least one medical service utilization in 2016; and the primary race was identified as White, Black, or Hispanic. Race was identified using the information provided by CMS. Information regarding how race was identified (e.g., self-report) is not available. The single claim-based definition to identify pediatric-onset conditions has shown good accuracy using administrative claims data, with sensitivity of 99% and positive predictive value of 79% (Reeves et al., 2014).

### 2.3 Outcome measures

The three prevalent outcomes measures, osteoporosis, all-cause fracture, and osteoarthritis, were identified by using at least one claim in any position. Osteoporosis was identified as osteoporosis with (M80 family codes) or without (M81 family codes) current pathological fracture. Fracture was identified as osteoporosis with current pathological fracture (M80 family codes) or fracture of the cervical vertebra (S12 family codes), ribs, sternum, or thoracic spine (S22 family codes), lumbar spine or pelvis (S32 family codes), shoulder or humerus (S42 family codes), radius or ulna (S52 family codes), femur (S72 family codes), or tibia or fibula (S82 family codes). Osteoarthritis was identified as polyosteoarthritis (M15 family codes), hip osteoarthritis (M16 family codes), knee osteoarthritis (M17 family codes), first carpometacarpal joint osteoarthritis (M18 family codes), or other/unspecified osteoarthritis (M19 family codes). The single claim-based algorithm to identify osteoporosis, fracture, and osteoarthritis has shown excellent ability to identify these medical conditions, as evidenced by positive predictive values up to 92% (Leslie et al., 2011), 97% (Narongroeknawin et al., 2012), and 94% (Shrestha et al., 2016), respectively.

### 2.4 Covariates

Age, sex, race (White, Black, Hispanic), and state of residency were available for analysis. A variable indicating region of U.S. (West, Midwest, South, and Northeast) was constructed to account for differences in region which may influence the outcome measures, such as climate (e.g., sunlight exposure and vitamin D status) and cultural (e.g., activity, diet) factors. Other important socioeconomic status indicators (e.g., income, education) were not available. Further, data regarding severity of CP using common clinical measures (e.g., gross motor function classification system) are not available in administrative claims, and >70% of the cohort had “other” or “unspecified” CP, thus not allowing for stratification or statistical adjustment for the clinical subtypes of CP (e.g., spasticity/athetoid, hemiplegic). Since comorbidity with neurodevelopmental conditions increases the medical complexity of CP (Reid et al., 2018), dichotomous variables were constructed for the presence of common neurodevelopmental conditions (i.e., intellectual disabilities [F70-79 family codes), autism spectrum disorders [F840 and F843-49 family codes], and epilepsy [G40 family codes]). To get a proxy of overall health status, dichotomous variables were constructed for the presence of the following noncommunicable diseases: ischemic heart diseases (I20-22 and I24-25 family codes), cerebrovascular diseases (I60-69 family codes), hypertensive (I10-16 family codes) and other cardiovascular diseases (heart failure, I50 family codes; atherosclerosis, I70 family codes), diabetes (including type 1 and 2: E08-13 family codes), mood affective disorders (F30-39 family codes), anxiety disorders (F40-48 family codes), substance abuse disorders (abuse or dependence F10-16 and F18-19 family codes including alcohol, opioid, cannabis, sedative/hypnotic/anxiolytic, cocaine, other stimulant, hallucinogen, inhalant, and other psychoactive substance related disorders), chronic obstructive pulmonary disease (J41-44 family codes), chronic kidney disease as previously described (Muntner et al., 2015) (N03, N18, E112, I12-13, and R80 family codes), liver diseases (K70-77 family codes), and malignant cancer (C00—7B family codes).

### 2.5 Statistical analysis

Descriptive characteristics were summarized and racial differences in descriptive characteristics were examined using the independent *t*-test or Chi-square test. *P* ≤ 0.005 (two-tailed) was used to determine statistical significance for this large sample, as recommended by a coalition of methodologists to detect new discoveries (Benjamin et al., 2018; Ioannidis, 2018). To be consistent with the *p*-value threshold, 99.5% binominal confidence intervals (CI) were calculated for the prevalence estimates of neurodevelopmental and noncommunicable disease comorbidities as the sample proportion ± the margin of error using a z-value of 2.807.

Direct age-standardization (Age Standardization and Population Counts and National Center for Health Statistics, 2014) for osteoporosis, fracture, and osteoarthritis was performed for each racial group and by sex. The 2016 U.S. adult population was used as a standard population. The U.S. Census Bureau released a table on age (5-year age brackets) and sex composition in the U.S. for 2016 (Age and Sex Composition in the United States: 2016, 2018). In order to make use of the population table in 5-year age groups, it was assumed that age was evenly distributed within the 15–19 year age bracket. Therefore, since 6.8% of U.S. males were 15–19 years old, it was assumed that 2.72% males were 18–19 years old (6.8% x (2/5)). A similar approach was performed for females.

Multivariable logistic regression models were developed separately for women and men with the outcome as osteoporosis in one set of analyses, fracture in another set of analyses, and osteoarthritis in the final set of analyses. The primary exposure variable for all models was race (reference: White). Model 1 adjusted for age, U.S. region, and neurodevelopmental comorbidities. Model 2 adjusted for the variables in model 1 and all noncommunicable diseases noted above. The main effect of race was interpreted. Effect estimates were reported as odds ratios (OR) with 99.5% CI.

A sensitivity analysis was performed that required at least two claims on separate days to identify CP and all neurodevelopmental and noncommunicable disease comorbidities to determine the main effect of race on the outcome measures using the fully adjusted model.

Analyses were performed using SAS version 9.4 (SAS Institute, Cary, NC, USA).

## 3 Results

Unadjusted descriptive characteristics of 18–64 year olds with CP (*n* = 16,488) stratified by White (*n* = 13,334), Black (*n* = 2477), and Hispanic (*n* = 677) are presented in Table 1. White adults with CP were older than Black and Hispanic adults with CP and Black adults with CP were older than Hispanic adults with CP. There was a significant difference in the distribution of U.S. region by race (all *p* < 0.005) with 54.3% of Black adults with CP residing in the South and 35.0% of Hispanic adults with CP residing in the West. White adults with CP were more likely to have intellectual disabilities compared to Hispanic adults with CP (p < 0.005). White adults with CP had higher prevalence of mood affective disorders, anxiety disorders, and cancer compared to Black and Hispanic adults with CP, and hypertensive and other cardiovascular diseases compared to Hispanic adults with CP (all *p* < 0.005). Black adults with CP had higher prevalence of cerebrovascular diseases, diabetes, and chronic kidney disease compared to White adults with CP, and hypertensive and other cardiovascular diseases compared to White and Hispanic adults with CP (all p < 0.005).

**Table 1:** Descriptive characteristics of 18–64 year olds with cerebral palsy.

Descriptive characteristics	White	Black	Hispanic	p-Value
Sample size, n	13,334	2477	677	
Age, mean (SD)	46.3 (11.3)	43.1 (12.3)	37.2 (11.0)	<0.001
18–40 years, %	31.9	43.4	67.4	
41–64 years, %	68.1	56.6	32.6	
Sex, %				0.023
Women	46.8	45.7	41.7	
Men	53.2	54.3	58.4	
Region, %				<0.001
West	15.4	7.4	35.0	
Midwest	30.6	21.4	12.4	
South	32.3	54.3	34.1	
Northeast	21.8	16.8	18.5	


Comorbidities	% (99.5% CI)	% (99.5% CI)	% (99.5% CI)	p-value
Intellectual disabilities	41.1 (39.9, 42.3)	37.6 (34.9, 40.3)	33.2 (28.1, 38.3)	<0.001
Autism spectrum disorders	5.3 (4.8, 5.8)	4.0 (2.9, 5.1)	6.2 (3.6, 8.8)	0.012
Epilepsy	39.8 (38.6, 41.0)	36.1 (33.4, 38.8)	41.5 (36.2, 46.8)	0.001
Ischemic heart diseases	4.8 (4.3, 5.3)	5.6 (4.3, 6.9)	3.8 (1.7, 5.9)	0.103
Cerebrovascular diseases	6.3 (5.7, 6.9)	8.5 (6.9, 10.1)	6.8 (4.1, 9.5)	<0.001
Hypertensive and other cardiovascular diseases	39.4 (38.2, 40.6)	46.6 (43.8, 49.4)	29.1 (24.2, 34.0)	<0.001
Diabetes	13.8 (13.0, 14.6)	17.5 (15.4, 19.6)	16.3 (12.3, 20.3)	<0.001
Mood affective disorders	30.3 (29.2, 31.4)	19.1 (16.9, 21.3)	23.5 (18.9, 28.1)	<0.001
Anxiety disorders	28.4 (27.3, 29.5)	16.8 (14.7, 18.9)	22.8 (18.3, 27.3)	<0.001
Substance abuse disorders	2.9 (2.5, 3.3)	3.5 (2.5, 4.5)	3.6 (1.6, 5.6)	0.199
Chronic obstructive pulmonary disease	6.9 (6.3, 7.5)	5.7 (4.4, 7.0)	5.3 (2.9, 7.7)	0.033
Chronic kidney disease	4.9 (4.4, 5.4)	7.4 (5.9, 8.9)	4.4 (2.2, 6.6)	<0.001
Liver diseases	4.7 (4.2, 5.2)	3.7 (2.6, 4.8)	5.2 (2.8, 7.6)	0.062
Cancer	4.2 (3.7, 4.7)	2.7 (1.8, 3.6)	1.8 (0.4, 3.2)	<0.001

### 3.1 Osteoporosis

**Fig. 1:**
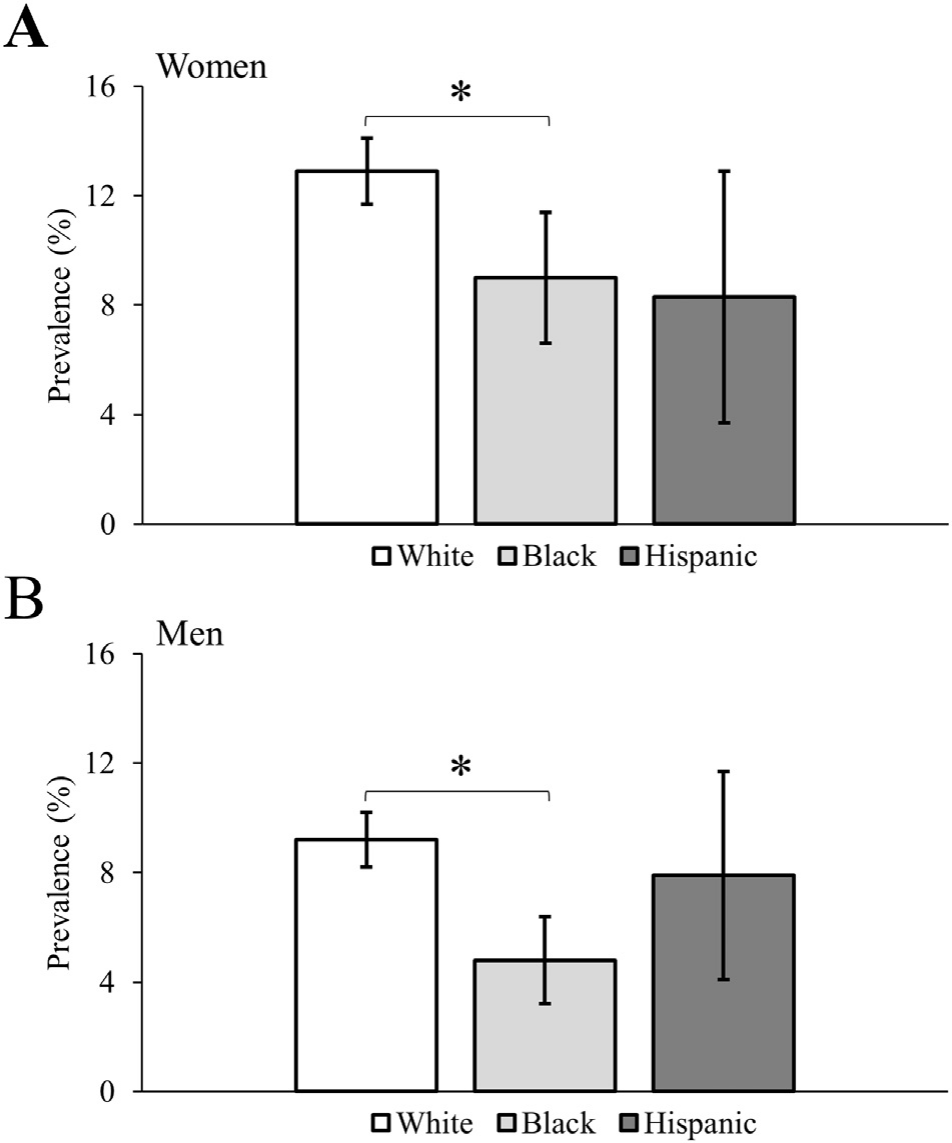
Age-standardized prevalence of osteoporosis for 18–64 years olds with cerebral palsy. Error bars represent 99.5% confidence intervals. *p-value < 0.005.

The age-standardized prevalence of osteoporosis by sex is presented in Fig. 1. White women and men with CP had higher prevalence compared to Black women and men with CP (both p < 0.005). No other statistical differences were observed across racial groups.

The adjusted odds for osteoporosis by sex is presented in Table 2. After adjusting for U.S. region and neurodevelopmental comorbidities (model 1) and compared to White adults with CP, the odds of osteoporosis was significantly lower for Black women (OR = 0.68; 99.5% CI = 0.49–0.93) and men (OR = 0.51; 99.5% CI = 0.35–0.74) with CP, but not for Hispanic women (OR = 0.61; 99.5% CI = 0.29–1.29) or men (OR = 0.61; 99.5% CI = 0.29–1.30) with CP. After further adjusting for all noncommunicable diseases (model 2), the odds were largely unchanged and remained significantly lower for Black women (OR = 0.66; 99.5% CI = 0.48–0.91) and men (OR = 0.51; 99.5% CI = 0.35–0.75) with CP.

**Table 2:** Multivariable logistic regression for osteoporosis among 18–64 year olds with cerebral palsy.

	Women		Men	
	Model 1 OR (99.5% CI)	Model 2 OR (99.5% CI)	Model 1 OR (99.5% CI)	Model 2 OR (99.5% CI)
Sample size, n	7657	7657	8831	8831
Race				
White	Reference	Reference	Reference	Reference
Black	0.68 (0.49, 0.93)	0.66 (0.48, 0.91)	0.51 (0.35, 0.74)	0.51 (0.35, 0.75)
Hispanic	0.61 (0.29, 1.29)	0.61 (0.29, 1.30)	0.88 (0.48, 1.62)	0.90 (0.49, 1.65)
Age (as continuous)	1.07 (1.06, 1.08)	1.06 (1.05, 1.08)	1.04 (1.03, 1.06)	1.05 (1.03, 1.06)
Region				
Northeast	Reference	Reference	Reference	Reference
West	0.50 (0.36, 0.70)	0.50 (0.36, 0.69)	0.71 (0.51, 0.98)	0.70 (0.50, 0.97)
Midwest	0.58 (0.45, 0.75)	0.59 (0.46, 0.77)	0.53 (0.40, 0.71)	0.53 (0.40, 0.71)
South	0.59 (0.46, 0.76)	0.59 (0.46, 0.76)	0.59 (0.45, 0.78)	0.59 (0.45, 0.78)
Intellectual disabilities	1.87 (1.52, 2.29)	1.91 (1.55, 2.35)	2.69 (2.13, 3.40)	2.70 (2.13, 3.42)
Autism spectrum disorders	1.48 (0.96, 2.27)	1.49 (0.97, 2.30)	0.75 (0.47, 1.19)	0.78 (0.49, 1.23)
Epilepsy	2.01 (1.64, 2.46)	1.96 (1.59, 2.40)	2.23 (1.78, 2.80)	2.20 (1.75, 2.76)
Ischemic heart diseases		1.25 (0.81, 1.92)		0.82 (0.51, 1.31)
Cerebrovascular diseases		1.17 (0.83, 1.66)		1.00 (0.68, 1.49)
Hypertensive and other cardiovascular diseases		1.09 (0.88, 1.35)		0.86 (0.68, 1.09)
Diabetes		0.93 (0.71, 1.22)		0.93 (0.68, 1.27)
Mood affective disorders		0.90 (0.72, 1.13)		0.74 (0.57, 0.97)
Anxiety disorders		1.01 (0.80, 1.27)		1.00 (0.77, 1.30)
Substance abuse disorders		1.16 (0.57, 2.33)		0.76 (0.35, 1.67)
Chronic obstructive pulmonary disease		1.46 (1.03, 2.07)		1.37 (0.93, 2.02)
Chronic kidney disease		0.98 (0.64, 1.48)		1.36 (0.88, 2.09)
Liver diseases		1.30 (0.84, 2.00)		1.34 (0.86, 2.09)
Cancer		1.58 (1.08, 2.31)		1.40 (0.87, 2.27)

OR, odds ratio; CI, confidence interval.

### 3.2 Fracture

**Fig. 2:**
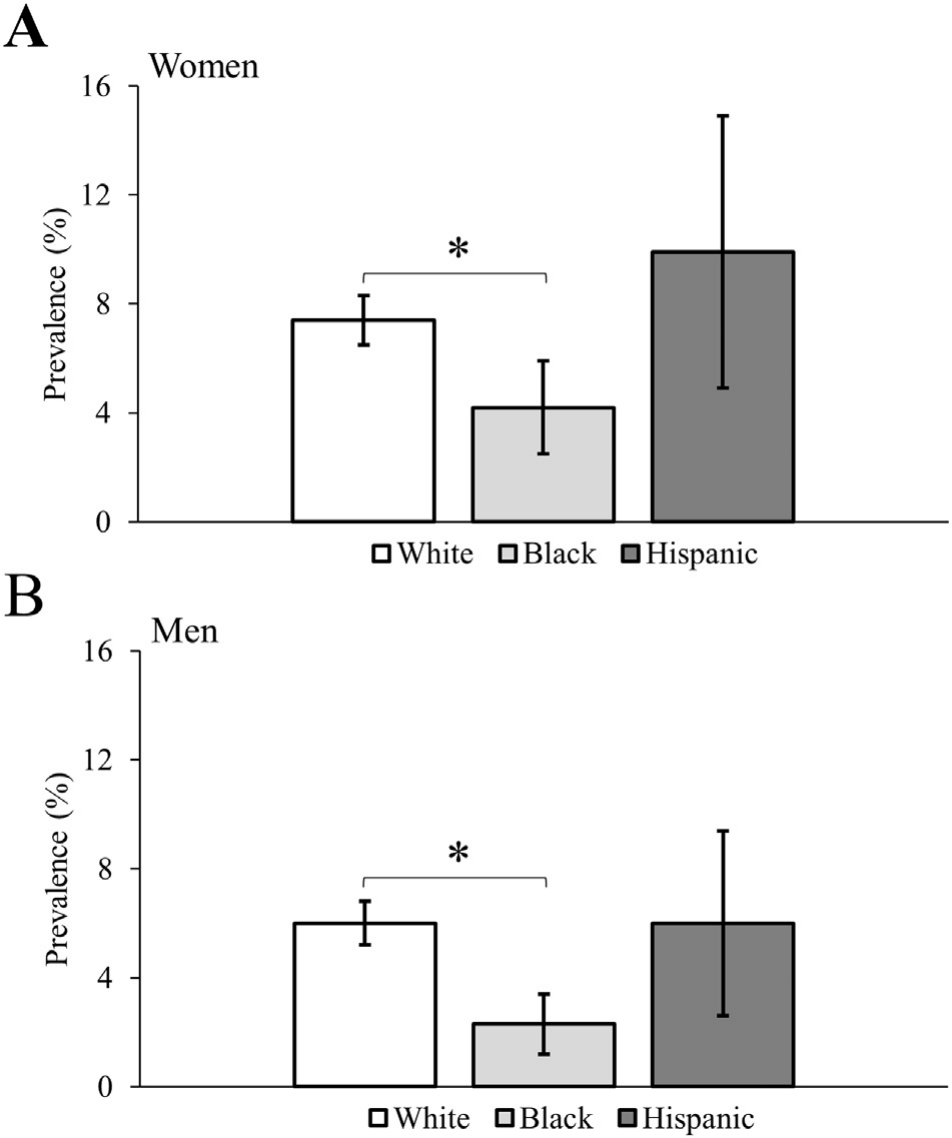
Age-standardized prevalence of fracture for 18–64 years olds with cerebral palsy. Error bars represent 99.5% confidence intervals. *p-value < 0.005.

The age-standardized prevalence of fracture by sex is presented in Fig. 2. White women and men with CP had higher prevalence compared to Black women and men with CP (both p < 0.005). No other statistical differences were observed across racial groups.

The adjusted odds for fracture by sex is presented in Table 3. After adjusting for U.S. region and neurodevelopmental comorbidities (model 1) and compared to White adults with CP, the odds of fracture was significantly lower for Black women (OR = 0.57; 99.5% CI = 0.37–0.88) and men (OR = 0.38; 99.5% CI = 0.22–0.65) with CP, but not for Hispanic women (OR = 1.09; 99.5% CI = 0.53–2.23) or men (OR = 0.87; 99.5% CI = 0.44–1.74) with CP. After further adjusting for all noncommunicable diseases (model 2), the odds were largely unchanged and remained significantly lower for Black women (OR = 0.57; 99.5% CI = 0.37–0.89) and men (OR = 0.39; 99.5% CI = 0.23–0.68) with CP.

**Table 3:** Multivariable logistic regression for fracture among 18–64 year olds with cerebral palsy.

	Women		Men	
	Model 1 OR (99.5% CI)	Model 2 OR (99.5% CI)	Model 1 OR (99.5% CI)	Model 2 OR (99.5% CI)
Sample size, n	7657	7657	8831	8831
Race				
White	Reference	Reference	Reference	Reference
Black	0.57 (0.37, 0.88)	0.57 (0.37, 0.89)	0.38 (0.22, 0.65)	0.39 (0.23, 0.68)
Hispanic	1.09 (0.53, 2.23)	1.10 (0.54, 2.26)	0.87 (0.44, 1.74)	0.85 (0.42, 1.70)
Age (as continuous)	1.03 (1.02, 1.05)	1.03 (1.02, 1.04)	1.02 (1.01, 1.03)	1.02 (1.01, 1.03)
Region				
Northeast	Reference	Reference	Reference	Reference
West	1.03 (0.67, 1.58)	1.08 (0.70, 1.67)	1.06 (0.70, 1.61)	1.07 (0.70, 1.62)
Midwest	1.35 (0.96, 1.91)	1.39 (0.98, 1.96)	0.94 (0.65, 1.35)	0.93 (0.64, 1.34)
South	1.18 (0.83, 1.67)	1.20 (0.84, 1.70)	0.89 (0.62, 1.27)	0.86 (0.60, 1.25)
Intellectual disabilities	1.21 (0.93, 1.56)	1.24 (0.95, 1.61)	1.18 (0.89, 1.56)	1.23 (0.93, 1.64)
Autism spectrum disorders	1.70 (1.03, 2.82)	1.64 (0.99, 2.74)	1.21 (0.72, 2.04)	1.13 (0.67, 1.92)
Epilepsy	1.40 (1.08, 1.80)	1.40 (1.08, 1.82)	1.25 (0.95, 1.64)	1.25 (0.94, 1.65)
Ischemic heart diseases		1.42 (0.86, 2.36)		0.99 (0.58, 1.68)
Cerebrovascular diseases		1.28 (0.83, 1.96)		1.25 (0.80, 1.96)
Hypertensive and other cardiovascular diseases		1.14 (0.87, 1.50)		1.07 (0.80, 1.44)
Diabetes		1.04 (0.74, 1.46)		1.05 (0.73, 1.52)
Mood affective disorders		1.23 (0.93, 1.62)		1.35 (1.00, 1.83)
Anxiety disorders		1.36 (1.03, 1.80)		1.50 (1.11, 2.02)
Substance abuse disorders		1.63 (0.83, 3.21)		2.55 (1.53, 4.25)
Chronic obstructive pulmonary disease		1.18 (0.76, 1.82)		1.28 (0.82, 1.99)
Chronic kidney disease		1.06 (0.64, 1.75)		1.02 (0.60, 1.76)
Liver diseases		0.87 (0.49, 1.55)		0.97 (0.55, 1.70)
Cancer		1.65 (1.04, 2.62)		0.49 (0.20, 1.19)

OR, odds ratio; CI, confidence interval.

### 3.3 Osteoarthritis

**Fig. 3:**
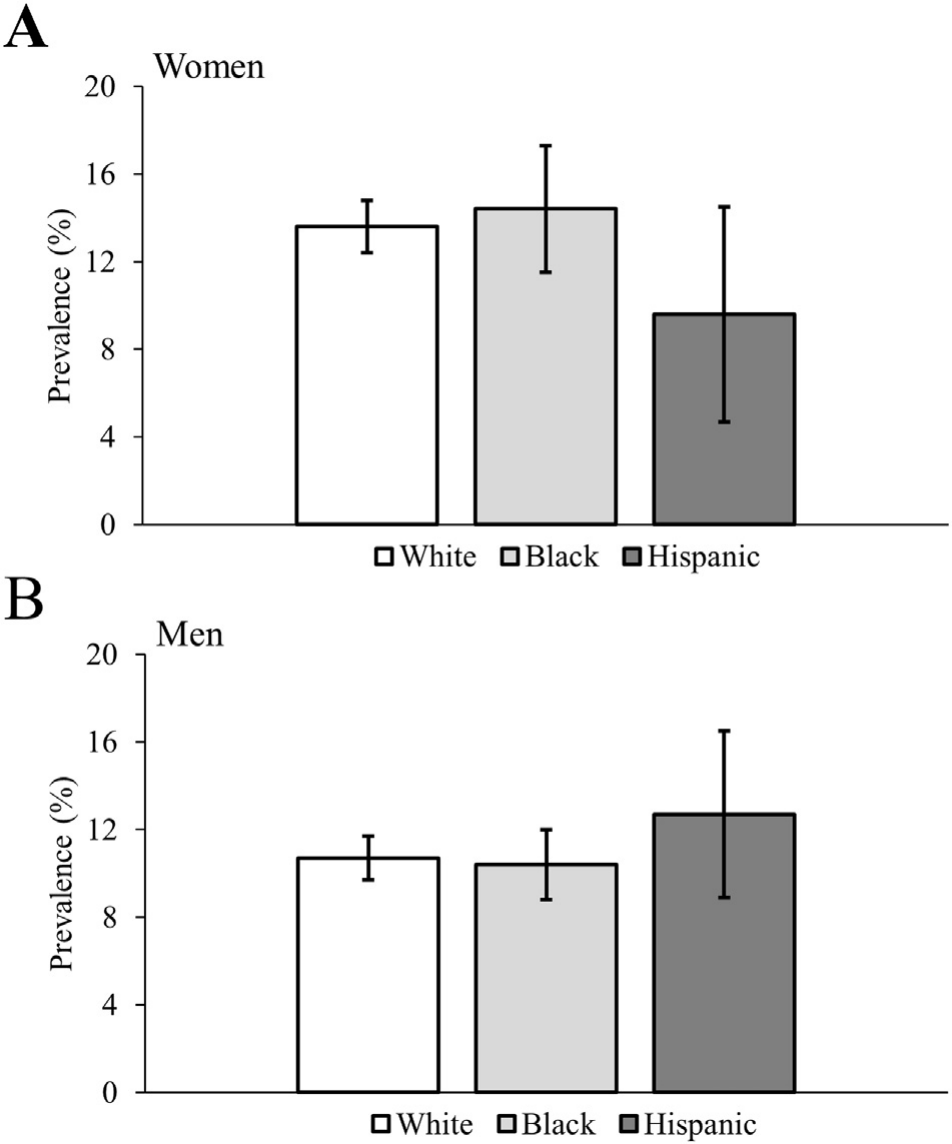
Age-standardized prevalence of osteoarthritis for 18–64 years olds with cerebral palsy. Error bars represent 99.5% confidence intervals. *p-value < 0.005.

The age-standardized prevalence of osteoarthritis by sex is presented in Fig. 3. No statistical differences were observed across racial groups.

The adjusted odds for osteoarthritis by sex is presented in Table 4. After adjusting for the variables in model 1 and 2, there was no difference in the odds of osteoarthritis for Black or Hispanic women or men with CP compared to White adults with CP.

**Table 4:** Multivariable logistic regression for osteoarthritis among 18–64 year olds with cerebral palsy.

	Women		Men	
	Model 1 OR (99.5% CI)	Model 2 OR (99.5% CI)	Model 1 OR (99.5% CI)	Model 2 OR (99.5% CI)
Sample size, n	7657	7657	8831	8831
Race				
White	reference	reference	reference	reference
Black	0.96 (0.74, 1.25)	0.97 (0.74, 1.29)	0.93 (0.71, 1.22)	0.93 (0.70, 1.24)
Hispanic	0.79 (0.42, 1.49)	0.79 (0.42, 1.49)	1.22 (0.74, 2.02)	1.14 (0.68, 1.90)
Age (as continuous)	1.06 (1.05, 1.07)	1.05 (1.04, 1.06)	1.06 (1.05, 1.07)	1.05 (1.04, 1.06)
Region				
Northeast	reference	reference	reference	reference
West	0.97 (0.71, 1.33)	1.07 (0.77, 1.47)	0.99 (0.71, 1.37)	1.01 (0.72, 1.41)
Midwest	1.23 (0.95, 1.58)	1.27 (0.98, 1.66)	1.26 (0.96, 1.66)	1.26 (0.95, 1.68)
South	1.19 (0.93, 1.53)	1.21 (0.93, 1.57)	1.34 (1.03, 1.75)	1.30 (0.99, 1.70)
Intellectual disabilities	0.69 (0.56, 0.84)	0.69 (0.56, 0.84)	0.86 (0.70, 1.05)	0.89 (0.72, 1.10)
Autism spectrum disorders	1.15 (0.71, 1.86)	1.10 (0.67, 1.81)	0.69 (0.42, 1.14)	0.63 (0.37, 1.05)
Epilepsy	0.88 (0.73, 1.08)	0.88 (0.72, 1.09)	1.00 (0.82, 1.22)	0.97 (0.79, 1.20)
Ischemic heart diseases		1.56 (1.06, 2.30)		1.32 (0.94, 1.86)
Cerebrovascular diseases		1.29 (0.93, 1.80)		1.25 (0.91, 1.72)
Hypertensive and other cardiovascular diseases		1.68 (1.37, 2.06)		1.45 (1.17, 1.79)
Diabetes		1.07 (0.84, 1.37)		1.28 (1.00, 1.64)
Mood affective disorders		1.55 (1.26, 1.90)		1.42 (1.14, 1.76)
Anxiety disorders		1.58 (1.28, 1.94)		1.38 (1.10, 1.72)
Substance abuse disorders		1.26 (0.74, 2.13)		1.81 (1.21, 2.71)
Chronic obstructive pulmonary disease		1.43 (1.04, 1.97)		1.49 (1.10, 2.02)
Chronic kidney disease		1.04 (0.71, 1.51)		1.23 (0.87, 1.74)
Liver diseases		1.69 (1.15, 2.47)		1.45 (1.00, 2.10)
Cancer		1.11 (0.75, 1.64)		1.36 (0.89, 2.08)

OR, odds ratio; CI, confidence interval.

### 3.4 Sensitivity analysis

The sensitivity analyses (*n* = 12,613; women, *n* = 5938; men, *n* = 6675) that required at least two claims to identify CP and all neurodevelopmental and noncommunicable disease comorbidities found the same conclusions as the primary analysis for racial differences in osteoporosis and fracture between White and Black women and men and no racial differences for osteoarthritis (data not shown).

## 4 Discussion

The main finding of this study is that there were racial differences in skeletal fragility but not osteoarthritis among young and middle-aged adults with CP. Specifically, even after adjusting for potential confounding factors, White women and men with CP had higher prevalence of osteoporosis and fracture compared to Black women and men with CP. Study findings significantly add to the growing body of literature by providing large, national-level data documenting racial differences in skeletal fragility, but not osteoarthritis, among adults with CP. This is important because there are substantial racial differences in non-CP populations for screening for skeletal fragility, osteoporosis diagnoses (Cheng et al., 2009), and osteoporosis-related treatment rates (Cauley, 2011; Hamrick et al., 2006). Therefore, understanding the differences in skeletal fragility and osteoarthritis across racial groups among adults with CP is a fundamental step towards developing successful interventions to optimize skeletal and joint health for this underserved population. Moreover, the prevalence estimates of osteoporosis, fracture, and osteoarthritis observed in this study were markedly elevated. Recent studies leveraging private insurance claims data from 2016 found that the general population without CP of the same age group had an osteoporosis prevalence of 1.3%, fracture prevalence of 2.7% (Whitney et al., 2019b), and osteoarthritis prevalence of 7.9% (Whitney et al., 2019c). Therefore, despite the low absolute prevalence (e.g., <15%), these estimates are still much higher than what would be expected for this younger age group. Importantly, early development of these conditions can significantly increase personal, disease, and economic burden (Burge et al., 2007; Tatangelo et al., 2019; Turkiewicz et al., 2014), and should be aggressively treated and prevented to minimize the cost to public health.

In the general population, White individuals, and particularly women, have greater risk of skeletal fragility compared to other racial groups (Cauley, 2011). However, mortality rate following a hip fracture is higher for Black women than White women (Jacobsen et al., 1992). Black individuals tend to have greater risk for osteoarthritis, but this association may be sex- and joint-specific and accounted for, at least in part, by confounding factors (Nelson et al., 2013; Wright et al., 2008). Further, Black individuals are less likely to receive hip or knee replacement than White individuals (Smith et al., 2017). Reasons for these racial differences in skeletal fragility and osteoarthritis have been attributed to differences in bone mineral density, geometric properties of bone and joints, geographic region, health disparities, and other socioeconomic factors (e.g., poverty) (Cauley, 2011; Wright et al., 2008; Nelson et al., 2016; Cauley et al., 2005; Tsai, 2019). Poor overall health status, as indicated by presence of noncommunicable diseases, is also associated with increased risk for skeletal fragility (Silverman et al., 2016). In the current study, the racial difference in skeletal fragility between Blacks and Whites remained even after accounting for U.S. region and comorbid neurodevelopmental conditions, which are each associated with skeletal fragility (Schrager et al., 2007; Neumeyer et al., 2017; Sheth et al., 2008). Further, the addition of all noncommunicable diseases to the model (i.e., model 2), which provided a proxy of overall health status, had little-to-no effect on the association of race with each outcome as evidenced by small or no deviations in the adjusted OR, suggesting a robust association between race and skeletal fragility among young and middle-aged adults with CP. However, this study was unable to directly assess other important indicators of socioeconomic status (e.g., income, education), CP severity, and physical activity levels, which are all associated with the outcomes. Whether there are differences in these measures among adults with CP and if they are associated with development of skeletal fragility or osteoarthritis requires further investigation.

There are many factors associated with the pathophysiology of skeletal fragility and osteoarthritis among individuals with CP. Whether and how these factors differ by racial group could provide insight into patient-specific care for skeletal fragility and osteoarthritis. Low levels of physical activity reported in children with CP predispose inadequate accrual of muscle and bone throughout growth and development (Whitney et al., 2017). Concomitant with reduced physical activity levels as these children age into and throughout their adult years (Day et al., 2007) is the emergence of a concerning musculoskeletal disease profile (Whitney et al., 2018c; Whitney et al., 2019b; O'Connell et al., 2019) that gets worse throughout the lifespan (Whitney et al., 2018d). There are also various skeletal deformities and malalignments of the lower extremities present among individuals with CP (Robin et al., 2008; Noonan et al., 2004; Miller et al., 1997), including femoral anteversion, hip subluxation, and joint dislocation. The musculoskeletal pathological phenotype in childhood is also governed by several CP-related factors (Chan and Miller, 2014), such as the type of CP (e.g., spastic, dyskinetic, ataxic), anatomical distribution of affected areas (e.g., hemiplegia, diplegia, quadriplegia), and the level of gross motor functional ability (e.g., independent ambulation, wheelchair user). Further, the degree to which muscles are affected (e.g., severity), how they are affected (e.g., spasticity), and the number of affected muscles and sites plays a foundational role on joint health by altering articular surface stresses during movement; all of which may lead to localized joint damage and increased risk for skeletal fragility and osteoarthritis.

In addition to the more established and recognized functional, anatomical, and physiological factors, epigenetics may have a unique or multiplicative role on the burden of skeletal fragility and osteoarthritis among individuals with CP, which is also influenced by race. Recent evidence suggests an altered epigenome between children with and without CP (Crowgey et al., 2018; Mohandas et al., 2018) and between preterm Black and non-Black infants (Salihu et al., 2016). Pre- and peri-natal factors and early life stressors can alter DNA methylation patterns in offspring (Roth et al., 2009) that remain sustained throughout the lifespan and are associated with chronic diseases later in life (Hao et al., 2018; Nieratschker et al., 2014). Unique DNA methylation profiles have been observed from tissue of postmenopausal women with osteoporosis (Reppe et al., 2017) and human osteoarthritic cartilage (Alvarez-Garcia et al., 2016) compared to their respective control group. Whether the causes of CP, subsequent poor psychosocial development (Whitney et al., 2019d; Whitney et al., 2018e; Whitney et al., 2019e), and race lead to DNA methylation patterns that predispose to early development of chronic diseases, including skeletal fragility and osteoarthritis, requires further investigation.

The limitations of this study must be discussed. First, CP-specific data (e.g., clinical subtypes and severity measures), other socioeconomic status indicators (e.g., income, education), and physical activity were not available or were inadequately coded in the CMS dataset, resulting in the inability to stratify or statistically adjust for these relevant constructs. In light of this limitation, this study adjusted for neurodevelopmental conditions that are commonly comorbid with CP and several high-burden noncommunicable diseases, which provides a proxy of the overall health status of the individual. Second, there is risk for unmeasured confounding which is inherent to observational research designs. E-values were therefore computed to determine the extent of unmeasured confounding (minimum strength of association with the exposure and outcome) needed to fully explain away a specific exposure-outcome association conditional on the set of covariates (Mathur et al., 2018; VanderWeele and Ding, 2017). Using the fully adjusted model for Black vs. White since Hispanic race was not different, the E-value (lower 95% CI) for women and men was 2.40 (1.43) and 3.33 (2.00) for osteoporosis, respectively, and 2.90 (1.50) and 4.57 (2.30) for fracture, respectively. Given the large E-values, it seems unlikely that unmeasured confounding largely biased these effect estimates for the exposure variable (i.e., Black vs. White). Third, the primary analysis used a single claim to identify CP and all neurodevelopmental and noncommunicable disease comorbidities. Validation studies have shown that two or more claims for a given medical condition improves sensitivity (Reeves et al., 2014; Kerr et al., 2000). However, single claim-based algorithms have shown a moderate-to-high positive predictive value (~80%) to detect pediatric-onset conditions (Reeves et al., 2014), such as CP, and a high positive predictive value for musculoskeletal medical conditions (Leslie et al., 2011); although, the accuracy of identifying conditions using claims data depends on the length of the study period (Leslie et al., 2011) and the medical condition examined (Reeves et al., 2014; Leslie et al., 2011; Doktorchik et al., 2019; Noyes et al., 2011). In light of this, a sensitivity analysis was performed that used at least two claims to identify CP and all comorbidities. The conclusion regarding racial differences for osteoporosis and fracture and no racial differences for osteoarthritis were the same as the primary analysis. Fourth, healthcare access and any-related inequalities were not assessed in the current study. Black and Hispanic individuals are historically underrepresented minorities that may not have the same resources for treatment and healthcare as do White individuals, which may have biased prevalence estimates for these groups to be slightly lower. Fifth, there was a considerable extent of other/unspecified osteoarthritis in the database, which prevented the ability to stratify results by location, such as hip or knee osteoarthritis. Sixth, the Hispanic group was much younger than the White or Black group and may consist of individuals that are White, Black, or mixed race, which may have biased results. However, two statistical methods accounted for age (i.e., direct age-standardization and age-adjusted logistic regression) and had the same conclusion. Future research is needed to determine if Hispanic in addition to White, Black, or other mixed race provides an additive or protective effect on skeletal fragility or osteoarthritis among adults with CP. Lastly, the current study did not have a comparison group which could have provided information as to whether the racial differences in outcomes are unique to adults with CP or are consistent with the general population. Future studies that leverage appropriate datasets are needed to examine a CP group by race interaction.

## 5 Conclusion

Young and middle-aged White individuals with CP have higher risk for skeletal fragility compared to Black individuals with CP. This study found no evidence of racial differences in osteoarthritis. While previous studies have recommended earlier screening strategies to detect musculoskeletal fragility for individuals with CP (Whitney et al., 2019b), study findings can assist development of detection and preventive services by considering the contribution of sex and race on skeletal fragility.
